# Self-Management Support After Burns: Protocol for a Multicenter, Stepped-Wedge Hybrid Type II Effectiveness-Implementation Study

**DOI:** 10.2196/86671

**Published:** 2026-03-16

**Authors:** Sharon L Blok, Marianne K Nieuwenhuis, Sonja M H J Scholten-Jaegers, Irma Visser, Raaba S M Thambithurai, Monique A C M de Craen-de Moor, Kiran C Baran, Anuschka S Niemeijer, Eelke Bosma, Corry K van der Sluis, Sven J G Geelen, National Burn Care, Education & Research Group, The Netherlands, Margriet E van Baar

**Affiliations:** 1 Alliance of Dutch Burn Care (ADBC), Burn Centre, Martini Hospital, van Swietenlaan 1, Groningen, 9728 NT, The Netherlands, 31 050 524 5245; 2 University of Groningen, University Medical Center Groningen, Department of Rehabilitation Medicine, Groningen, The Netherlands; 3 Research Group Healthy Ageing, Allied Health Care and Nursing, Hanze University of Applied Sciences, Groningen, The Netherlands; 4 University of Groningen, University Medical Center Groningen, Department for Human Movement Sciences, Groningen, The Netherlands; 5 Dutch Association of Burn Survivors, Beverwijk, The Netherlands; 6 Alliance of Dutch Burn Care (ADBC), Burn Centre, Maasstad Hospital, Rotterdam, The Netherlands; 7 Erasmus School of Health Policy and Management, Erasmus University Rotterdam, Rotterdam, The Netherlands; 8 Alliance of Dutch Burn Care (ADBC), Burn Centre, Red Cross Hospital, Beverwijk, The Netherlands; 9 Martini Hospital, Department of Surgery, Groningen, Groningen, The Netherlands; 10 See Acknowledgments

**Keywords:** burns, burn survivor, self-management, self-management support, stepped-wedge study, RE-AIM, hybrid type II, implementation science, Reach, Effectiveness, Adoption, Implementation, and Maintenance

## Abstract

**Background:**

After a burn injury, the survivors have to manage and integrate the physical, psychological, and social consequences of their injury into their daily lives, such as functional limitations, aesthetic complaints, and fatigue. How successful survivors of burn injuries are at this depends on their self-management skills. Health care professionals play an important role in supporting the self-management of survivors of burn injuries. Currently, there are no burn-specific self-management support interventions. Therefore, we developed a self-management support intervention for survivors of burn injuries, called BreeZe (Brandwonden en Zelfmanagement).

**Objective:**

This study aimed to describe a study protocol to implement and evaluate the BreeZe intervention.

**Methods:**

This multicenter study in the Netherlands is an implementation-effectiveness hybrid type 2 study, with a nonrandomized stepped-wedge design. Starting April 2024, 3 phases have been sequentially rolled out across the 3 specialized Dutch burn centers over a period of 20 weeks—the preimplementation phase (usual care), implementation phase, and postimplementation phase. To identify barriers and facilitators of implementation, the Consolidated Framework for Implementation Research (CFIR) will be used. For evaluation, the Reach, Effectiveness, Adoption, Implementation, and Maintenance (RE-AIM) evaluation framework is used. The coprimary outcomes are (1) self-management skills and (2) the implementation outcomes are reach, adoption, implementation, and maintenance. Secondary effectiveness outcomes are self-regulation, participation, dependency, patient-centeredness for survivors of burn injuries, self-management support skills for health care professionals, and cost-effectiveness. Data collection for survivors of burn injuries occurs at 2 weeks, 6 months, and 12 months post discharge, using questionnaires. Data collection for health care professionals occurs before training and 3, 6, and 12 months post implementation, using questionnaires, video observations, and interviews. Data analysis will include both quantitative and qualitative methods for comprehensive evaluation.

**Results:**

Participant recruitment ended on June 30, 2025. Follow-up data collection ends in July 2026.

**Conclusions:**

This study will evaluate both the effectiveness and implementation of the BreeZe self-management support intervention for survivors of burn injuries using a hybrid effectiveness–implementation design. By applying the CFIR and RE-AIM frameworks within a stepped-wedge design embedded in routine burn aftercare, this study aims to generate robust and practice-relevant evidence on how self-management support can be effectively implemented in burn care. The findings are expected to inform both clinical practice and future implementation efforts in burn aftercare settings.

## Introduction

### Background and Rationale

After a burn injury, the survivors have to integrate numerous short- and long-term consequences of their injury in their daily lives. These consequences extend beyond the acute phase of care and may persist for many years after the initial injury, resulting in ongoing physical, social, and emotional challenges. For example, scar maturation of burn scars is a slow process with multiple phases that can take up to 2 years or longer [1]. Other common long-term consequences include chronic pain, pruritus, reduced physical functioning, and enduring psychosocial difficulties, such as emotional distress and challenges related to altered appearance and social participation. All these long-term consequences frequently require ongoing monitoring, adaptation, and behavioral management by the survivor of burn injuries. Due to the persistent nature of these consequences and the associated long-term care demands, burn injuries have increasingly been conceptualized as a chronic condition rather than a purely acute event [2]. In order to manage the long-term and often persistent consequences of a burn injury and integrate these into daily life, survivors of burn injuries need to actively engage in self-management [3-6].

Self-management is a comprehensive, multifaceted process, which Richard and Shea [7] defined as “the ability of the individual in conjunction with family, community, and health care professionals to manage symptoms, treatments, lifestyle changes, and psychosocial, cultural, and spiritual consequences of health conditions.’’ This definition emphasizes that self-management is not restricted to the medical domain but encompasses all aspects of life. For survivors of burn injuries, self-management therefore involves managing a range of ongoing demands, including persistent physical symptoms (such as pain, itch, or fatigue), adherence to often long-term wound and scar care regimens, psychosocial adjustment to changes in appearance and functioning, and the integration of these demands into everyday routines and social participation [8]. Although the relative importance and manifestation of these demands differ between individuals, survivors of burn injuries are commonly required to simultaneously manage these consequences over an extended period of time. Given the multimodality of these consequences over a long period of time, self-managing the consequences of burns is a complex and challenging process for survivors of burn injuries [8-11].

Although self-management is primarily a personal process, survivors of burn injuries can be supported in this process by health care professionals. Self-management support can be defined as the systematic provision of education and supportive interventions to improve patients’ skills and confidence in managing their health problems, including regular assessment of progress and problems, goal setting, and problem-solving support [12]. In burn aftercare, such support is particularly relevant because responsibility for managing ongoing physical, treatment-related, and psychosocial consequences shifts increasingly to the survivor of burn injuries after discharge, when professional contact becomes less frequent. Previous research has shown that interventions focused on self-management support may lead to improved patient self-management skills and related outcomes, such as enhanced quality of life [5,13]. These interventions are typically composed of several elements, such as education, goal-setting, and behavioral coaching [14,15]. Although many components of self-management support interventions are not disease-specific, tailoring interventions to the specific demands and context of the target population is considered a core principle of effective self-management support [16].

Based on our review of the current literature on burn aftercare and self-management support interventions, we identified no published interventions that were specifically developed for survivors of burn injuries during their aftercare trajectory (SL Blok, unpublished data). Given the diverse yet recurring self-management demands faced by survivors of burn injuries during aftercare, we developed a self-management support intervention for Dutch survivors of burn injuries called BreeZe (Brandwonden en Zelfmanagement) [17]. The overall goal of the BreeZe intervention is to enhance the self-management skills of survivors of burn injuries in order to integrate treatment and life goals and subsequently optimize the quality of life of survivors of burn injuries and health-related outcomes. The BreeZe intervention is based on the content of the ZENN (Zelfmanagement Na Niertransplantatie) intervention, an evidence-based self-management support intervention for kidney transplant recipients, which we adapted to the context of burn care [18]. The ZENN intervention was based on the theoretical framework of the self-regulation theory [19]. The main intervention strategies of ZENN, and therewith BreeZe, are based on evidence-based techniques, namely goal setting and pursuit, solution-focused brief therapy, and motivational interviewing [20-22]. In practice, this means that the intervention focuses on a positive approach in order to enhance the intrinsic motivation and self-efficacy of survivors of burn injuries to encourage sustainable behavior change regarding self-management in burns aftercare. To achieve this, we will implement the BreeZe intervention using various implementation strategies. These will include educating health care professionals on self-management support, training them in communication skills (eg, motivational interviewing), and providing supportive materials, such as a decision aid tool (ie, self-management web) and a workbook tailored to survivors of burn injuries [17,18].

### Objectives

In this paper, we provide insight into the study protocol that we aim to use to evaluate the BreeZe intervention in Dutch burn care. For more detailed information about the development of the BreeZe intervention, including preliminary feedback regarding acceptability and feasibility, we refer readers to a separate article by Geelen et al [17].

The coprimary objectives of the evaluation study are (1) the effectiveness of the BreeZe intervention in improving self-management skills in survivors of burn injuries, and (2) the effects of our implementation approach on the implementation outcomes reach, adoption, implementation, and maintenance. The secondary objectives will be to evaluate the effectiveness of the BreeZe intervention on more latent health outcomes (eg, return to work or school) and to assess the cost-effectiveness of the BreeZe intervention from a societal perspective.

## Methods

### Trial Design

This multicenter stepped-wedge hybrid type II effectiveness-implementation study is designed to test the effectiveness of the BreeZe intervention while also testing the effect of our implementation approach [23,24]. The study is structured within a superiority framework, meaning we will systematically compare outcomes between the control and intervention groups, as well as assess changes from baseline to follow-up.

All 3 specialized Dutch burn centers will participate in this study—Martini Hospital Groningen (center 1), Red Cross Hospital Beverwijk (center 2), and Maasstad Hospital Rotterdam (center 3).

This study has 3 phases and uses in all 3 phases the implementation science frameworks “Consolidated Framework for Implementation Research” (CFIR) and the “Reach, Effectiveness, Adoption, Implementation, and Maintenance” (RE-AIM) [25-27].

In the first phase, the preimplementation phase, we will use the CFIR to provide insight into the context-specific determinants for implementing the BreeZe intervention, thereby informing the development of a context-specific implementation approach. Meetings will be held with burn physicians, nurse practitioners, aftercare nurses, and burn center managers. The findings will build on the preliminary feedback regarding acceptability and feasibility received during the development of the BreeZe intervention [17]. In addition, during the preimplementation phase, we will recruit patients to form a control group to evaluate effectiveness. This preimplementation phase starts for all 3 specialized burn centers in April 2024. Although protocol registration was finalized after the start of data collection in the preimplementation phase, we confirm that no modifications to predefined outcomes or analyses were made based on data collected before registration.

**Figure 1.**
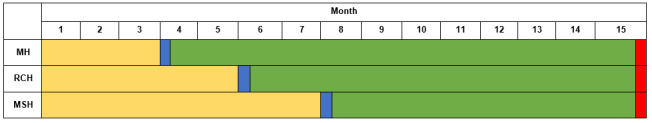
Overview of the stepped-wedge design (yellow represents preimplementation phase, blue represents implementation phase, green represents postimplementation phase, and red represents end of postimplementation phase). MH: Martini Hospital; MSH: Maasstad Hospital; RCH: Red Cross Hospital.

The second phase, the implementation phase, introduces and implements the BreeZe intervention for which we will use a stepped-wedge approach, visualized in Figure 1. We will begin by introducing the BreeZe intervention using the context-specific implementation approach developed in the preimplementation phase at center 1, in June 2024. Next, we will introduce the BreeZe intervention in center 2 in September 2024 and in center 3 in November 2024, respectively. Based on our development of the BreeZe intervention, we recognize that this implementation phase will require, at a minimum, education and training of health care professionals on how to apply the BreeZe intervention in routine clinical care. We anticipate that this implementation phase, consisting of training and intervention initiation, will take approximately 1 week; the intervention will then be delivered continuously.

In the third and final phase, each burn center transitions to the postimplementation phase. Health care professionals are asked to apply the BreeZe intervention in routine clinical care. In addition, we will recruit patients during the postimplementation phase for the evaluation of the effectiveness, and we will collect data to assess the effect of our implementation approach using the RE-AIM. This will be our intervention group. Recruitment of patients ended on July 1, 2025.

### Study Setting and Participants

#### Burn Centers

All 3 specialized burn centers in the Netherlands are characterized by their highly specialized multidisciplinary teams and are involved in the acute care of survivors of burn injuries, as well as in the aftercare. These centers, along with specialized trauma centers and other nonburn facilities, collectively constitute a well-organized network for burn care. Burn aftercare involves regular outpatient appointments, focused on wound and scar management, minimizing complications, and long-term rehabilitation. The 3 centers are similar in most factors, with a few minor differences. There is a small difference in the geographical catchment area, with Groningen covering a larger but less densely populated region. All centers provide both inpatient and outpatient burn care, but there is a difference in the organizational structure of burn aftercare, particularly with respect to the professional roles involved and the level of autonomy in outpatient follow-up. Nevertheless, the characteristics of the patient populations between the centers are generally comparable, as the Netherlands is a small country.

#### Patients With Burns

The Emergency Management of Severe Burns referral criteria guide the decision-making process to refer patients to a specialized burn center [28]. Criteria for survivors of burn injuries to be treated in one of the specialized burn centers are (1) a total burned body surface area of >10%; (2) >5% full-thickness burns; (3) circular burns around the neck, thorax, or extremities; (4) burns around functional areas; (5) burns accompanied by other trauma; (6) chemical burns; (7) preexisting disease; or (8) burns due to electricity. Patients meeting the Emergency Management of Severe Burns criteria are either directly admitted to a burn center or subsequently transferred. In the Netherlands, around 1000 patients with burns are admitted to one of the 3 specialized burn centers per year (*Dutch Burn Repository R3*). Minor burn injuries can be managed in one of the burn centers by outpatient consultations, but can also be managed in specialized tertiary trauma centers or other nonburn facilities.

Survivors of burn injuries will be recruited continuously throughout each stepped-wedge study period. Only newly referred survivors of burn injuries entering burn aftercare at the time of study initiation may be approached for participation, provided they meet the eligibility criteria.

#### Health Care Professionals

The health care professionals involved in burn aftercare are surgeons, burn physicians, nurse practitioners, aftercare nurses, burn care nurses, physical therapists, occupational therapists, social workers, and psychologists.

### Eligibility Criteria

#### Survivors of Burn Injuries

Eligible for inclusion are patients with burns who receive burn aftercare at 1 of the 3 specialized burn centers, who (1) are 18 years of age or older, (2) have been admitted for more than 24 hours or undergoing a wound-management procedure in theater, and (3) have proficiency in the Dutch language, as judged by the treating clinician. Exclusion criteria are cognitive limitations in such a way that self-management skills cannot be learned or executed, an acute psychiatric illness, or being discharged to a different health care institution. Cognitive limitations and acute psychiatric illness will be assessed by the treating clinician based on routine clinical judgment.

#### Health Care Professionals

Depending on the burn center and the complexity of the patient, a burn physician, nurse practitioner, or aftercare nurse is assigned the role of care coordinator in burn aftercare, putting them in the position to systematically provide self-management support. Therefore, all nurse practitioners, aftercare nurses, and burn physicians will be invited to participate and receive training.

### Intervention

#### Explanation for the Choice of Comparators

The selection of comparators in this study is based on the need to comprehensively evaluate whether the BreeZe intervention is effective. Usual care serves as an appropriate comparator, as it reflects current burn aftercare practices, and it provides a baseline for assessing the added value of the BreeZe intervention.

#### Intervention Description

##### BreeZe—Overall Goal

The overall goal of BreeZe is to enhance the self-management and self-regulation skills of survivors of burn injuries, allowing them to integrate treatment and life goals and subsequently optimize their quality of life, participation in society, and health-related outcomes. The BreeZe intervention is based on the content of the ZENN intervention, an evidence-based self-management support intervention for recipients of a kidney transplant [18], which we adapted to the context of burn care [17]. The ZENN intervention was based on the theoretical framework of the self-regulation theory [19]. The main intervention strategies of ZENN, and therewith BreeZe, are based on evidence-based techniques, namely goal setting and pursuit, Solution-Focused Brief-Therapy, and motivational interviewing [20-22,29]. In practice, this means that the BreeZe intervention focuses on a positive approach in order to enhance the intrinsic motivation and self-efficacy of survivors of burn injuries to encourage sustainable behavior change regarding self-management.

##### BreeZe—Practical Application at Patient Level

The start of BreeZe depends on the health care trajectory of the survivor of burn injuries. For survivors of burn injuries who were admitted for a 1-day stay at a burn center for a debridement or skin graft surgery, BreeZe will commence ±7 to 14 days after discharge, and the steps of BreeZe will be divided into 4 sessions. For survivors of burn injuries who were admitted for more than 24 hours at a burn center, an extra session is added, which will take place around the day of discharge. It is important to note that the number of outpatient consultations needed to progress through these sessions depends on the needs for self-management support of the survivor of burn injuries and the logistical constraints of the burn center. Hence, a fixed intervention timeline cannot be specified. In case one or more informal caregivers are involved, they will also be offered the opportunity to be engaged from the start.

In the initial session, a holistic review of how the patient is doing in 14 life areas takes place, as outlined in the communication aid used, the self-management web. The self-management web is not a measurement tool, but is intended to structure the conversation, to open the view of both survivors of burn injuries and health care professionals to discuss possible topics, and to make this visual for the patient. This web can also be revisited in subsequent sessions to make progress on personal goals, visual and tangible. This tool is also suitable for patients who have lower health literacy or language skills [18]. After completing the self-management web, the health care professional stimulates the survivor of burn injuries to prioritize a life area and to address and set a SMART (Specific, Measurable, Attainable, Relevant, and Time-based) goal. A global plan of action for goal attainment will be agreed upon. In addition, motivation for change and self-efficacy of survivors of burn injuries in relation to the goal will be discussed using visual analog scales. To support this process, a booklet has been developed that visually represents the overall BreeZe process and provides space to document all goals and progress. The second and third sessions are used to evaluate the progress of goal attainment. Facilitators and barriers are explored, and if necessary, strengths are emphasized, progress is complimented, and the action plan may be revised. In addition, motivation and self-efficacy will be evaluated and encouraged, alongside a discussion of internal versus external attribution of successes. This means that the survivor of burn injuries will be stimulated to look at his or her own role in the success, to promote internal attribution of that success. The aim of the fourth session is to evaluate and discuss the goal attainment, relapse prevention, and generalization of the learned skills to other situations.

For survivors of burn injuries who are discharged home after burn center admission and thereafter receive outpatient burn care, a session will be added to BreeZe to ensure they have a positive hospital-to-home experience. Adding this session to Breeze is pertinent to its success as survivors of burn injuries and their informal caregivers may feel overwhelmed, unsupported, and insecure during the hospital-to-home transition [30], which in turn strongly affects the ability to self-manage post discharge [18,31,32]. To counter possible negative perceptions, survivors of burn injuries and their informal caregivers have to feel empowered, encounter empathy and expertise, and have their expectations managed during the transition from hospital to home [18,30]. For these survivors of burn injuries, BreeZe will commence before discharge from the burn center. In this first session, the health care professional working in the burn aftercare visits the survivor of burn injuries in the burn center to discuss and prepare for the first 14 days post discharge. Instead of merely providing information to ensure understanding, motivation, and skills of survivors of burn injuries, these health care professionals will be trained to reach a common ground of understanding with the patient on what is needed during the first 14 days post discharge. Once this common ground has been reached, the health care professional uses solution-focused communication techniques to discuss the desired outcome at 14 days post discharge, discuss self-efficacy, and agree on a global plan of action for the first 14 days post discharge. A follow-up call ±3 days post discharge will allow the health care professional to explore the patient’s actions, questions, and misunderstandings, including discrepancies in the discharge plan to be identified and addressed, as well as any concerns from (informal) caregivers.

The BreeZe sessions will primarily be delivered in-person during routine outpatient burn aftercare appointments at the burn center. Where clinically appropriate and in line with routine care practices, sessions may also be delivered by telephone. The intervention does not include fully digital delivery, but supporting materials (eg, workbook and decision aids) are provided in paper format to facilitate continuity between sessions. This flexible delivery mode is intended to support the feasibility and integration of the intervention within existing burn aftercare workflows.

For a detailed description of the BreeZe intervention components and the aims of each session, we refer to the intervention development study by Geelen et al [17].

### Criteria for Discontinuing or Modifying Allocated Interventions

Participants can withdraw their permission to participate at any given time without providing a reason. Health care professionals will ensure that withdrawal of a participant does not influence their treatment quality.

### Strategies to Improve Adherence to Interventions: The BreeZe Implementation Approach

We will use the CFIR to develop a context-specific implementation approach for the BreeZe intervention and guide its application in each burn center [25]. Our main implementation strategies include education and training of health care professionals, enabling them to deliver the BreeZe intervention. This education and training will feature a blended approach that incorporates an electronic learning (e-learning) followed by an in-person training session.

The e-learning is divided into 3 parts, blending theory with interactive questions and videos. Part 1 features 2 short webinars. The first covers the key components and theoretical basis of self-management support, while the second focuses on the solution-focused conversation method and the introduction of the self-management web tool. Part 2 explains the structure and phases of providing self-management support through the BreeZe intervention with example videos. Finally, part 3 presents a practical application through a challenging case study of a patient struggling with a chronic condition, such as burns.

The content of the in-person training session builds on the e-learning. The in-person training session focuses on the practical application of the BreeZe intervention in routine clinical care. Burn physicians, aftercare nurses, and nurse practitioners will learn to activate survivors of burn injuries to generate their own solutions rather than focusing on the problems. They will also learn how to support survivors of burn injuries in achieving progression toward their goals in a short space of time. The training will consist of 3 blocks of 2 hours given on the same day and will be provided by a psychologist (Emma Massey, PhD) together with a training actor. Dr Massey was involved in the development and implementation of the ZENN intervention on which BreeZe is based [18]. In addition, there will be an online booster session after 2 months with the aim of discussing what the health professionals have encountered in their practice and optimizing standardization between health care professionals.

In addition to education and training, we will hold frequent meetings in the preimplementation phase with burn physicians, nurse practitioners, aftercare nurses, and burn center managers to identify context-specific determinants for implementing the BreeZe intervention in each burn center. The CFIR-ERIC (expert recommendations for implementing change) Matching Tool will help us translate these determinants into implementation strategies supporting the education and training [25,33]. Together, they form the implementation approach, which will be put into practice during the implementation phase, that will improve adherence to the BreeZe intervention.

### Relevant Concomitant Care Permitted or Prohibited During the Trial

During the preimplementation phase, health care professionals provide aftercare as usual. No other concomitant care or interventions are prohibited during the study.

### Outcomes

#### Overview

To translate our intervention into practice, we adhere to the RE-AIM framework [26,27]. The RE-AIM framework consists of 5 dimensions—reach, effectiveness, adoption, implementation, and maintenance. We have presented each outcome measure under its RE-AIM dimension in Table 1. Detailed information on the definitions of the RE-AIM dimensions and their related measures can be found in Multimedia Appendix 1.

**Table 1.** Overview of the outcome measures categorized by each RE-AIM^a^ dimension.

Outcome	Instrument	Number of questions	Number of domains	Answer scale	Minimum-maximum score
Coprimary outcome effectiveness measure					
Self-management skills	Partners in Health scale [34]	12	2	9-point Likert scale	0‐96
Secondary outcome effectiveness measures – survivor of burn injuries level					
Self-regulation	Self-Regulation Assessment [35]	22	4	5-point Likert scale	0‐110
Participation	Measure of Experiential Aspects of Participation [36]	4	4	5 answer options	—^b^
Daily activities	EuroQol-5D-5L [37]	1	—	5-point Likert scale	1‐5
Self-care	EuroQol-5D-5L [37]	1	—	5-point Likert scale	1‐5
Dependency	New formulated question	1	—	5-point Likert scale	1‐5
Return to work or school	Adjusted International Consortium for Health Outcomes Measurement [38]	3	—	5 answer options	—
Patient centeredness	American Consumer Assessment of Health Plan Survey [39]	5	—	4-point Likert scale	0‐20
Secondary outcome effectiveness measures – health care professional level					
Self-management support skills	Self-Efficacy and Performance in Self-management Support [40]	36	6	5-point Likert scale	0‐180
Need-supportive counseling	Coding and Observing Need-Supportive Counseling in Chronic Care Encounters [41]	44	9	5-point Likert scale	0‐24
	Nurse-Child Interaction Taxonomy [42]	16	3	Percentages	0%‐100%
Secondary outcome effectiveness measures – setting level					
Cost-effectiveness	EuroQol-5D-5L [37]	6	5	5-point Likert scale Visual Analog Scale	5‐25 0‐10
Coprimary outcome implementation approach measures					
Reach	Dutch Burn Repository R3	—	—	Frequencies and percentages	—
Reach	Project coordinator records	—	—	—	—
Reach	Screening form	—	—	—	—
Reach	CFIR^c^-informed interviews	—	—	—	—
Adoption (staff level)	CFIR-informed interviews	—	—	—	—
Adoption (setting level)	Project coordinator records	—	—	—	—
Intervention fidelity	Electronic patient records	—	—	Frequencies and percentages	—
Intervention fidelity	Project coordinator records	—	—	—	—
Intervention fidelity	CFIR-informed interviews	—	—	—	—
Implementation fidelity	Completion of implementation plan	—	—	Frequencies and percentages	—
Implementation adaptations	Framework for Reporting Adaptations and Modifications-Enhanced [43]	—	—	—	—
Maintenance (staff level)	Provider Report of Sustainment Scale [44]	3	—	Frequencies and percentages	—
Maintenance (staff level)	Self-Efficacy and Performance in Self-management Support [40]	36	6	5-point Likert scale	0‐180
Maintenance (staff level)	CFIR-informed interviews	—	—	—	—
Maintenance (setting level)	Clinical Sustainability Assessment Tool [45]	35	7	7-point Likert scale	0‐245
Maintenance (setting level)	CFIR-informed interviews	—	—	—	—

a RE-AIM: Reach, Effectiveness, Adoption, Implementation, and Maintenance.

b Not applicable.

c CFIR: Consolidated Framework for Implementation Research.

#### RE-AIM: Reach

To determine the representativeness of survivors of burn injuries, participant demographics will be compared with those of nonparticipants using the Dutch Burn Repository R3. Nonparticipants will include both survivors of burn injuries who did not meet the eligibility criteria and eligible survivors of burn injuries who declined participation. We will compare some characteristics, such as sex, age, total body surface area burned, operation, and length of stay. Comparisons will be descriptive in nature and will be used to assess the representativeness of the study sample and to explore potential selection effects, rather than to draw causal inferences.

#### RE-AIM: Effectiveness

##### Effectiveness on Health Outcomes of Survivors of Burn Injuries – Coprimary Outcome Measure

Self-management skills: The primary outcome measure is self-management skills, which will be assessed with the revised version of the Partners in Health scale (PiH) [34]. The PiH measures self-management behavior. In the Dutch PiH, two subscales are identified, (1) knowledge and coping, and (2) recognition and management of symptoms and adherence to treatment [46]. Higher scores indicate better self-management skills. Psychometric properties of the PiH scale show good construct validity and reliability. Cronbach α to measure internal consistency varies from 0.82 to 0.86 [34,46,47]. The intraclass correlation coefficient (ICC) to measure test-retest reliability varies from 0.77 in the French version PiH to 0.818 in the Chinese version of the PiH, indicating a good test-retest reliability [48,49]. Although no data have been found on responsiveness, the PiH has shown to provide valid and consistent evidence of self-management skills of patients with chronic conditions [47].

##### Effectiveness on Health Outcomes of Survivors of Burn Injuries – Secondary Outcome Measures

Self-regulation: Currently available self-report measures of self-management among patients with chronic conditions do not address all elements incorporated into the intervention and are generic. Therefore, Mol et al [35] developed a new self-report instrument (Self-Regulation Assessment [SeRA]) based on a model of self-regulation, expert and patient opinions, and cognitive interviews. The SeRA assesses four domains of self-regulation: (1) insight into one’s own health condition, (2) insight into own capabilities, (3) trust in self and apply self-regulation, and (4) organization of help. A higher score on the scale indicates a higher level of self-regulation skills in the context of an organ transplantation. Mol et al [35] showed that the SeRA exhibits high internal consistency (Cronbach α=0.93) and that it is a valid and reliable instrument to assess the necessary self-regulation skills among rehabilitation patients.

Participation: To assess participation, we will use a shortened version of the Measure of Experiential Aspects of Participation [36]. Participants will be asked if they participate in four different life domains, (1) employment, (2) mobility, (3) sport, and (4) exercise. The response options will be used to assess both participation in the activity and the individual’s satisfaction with the level of participation.

Daily activities: Participants will be asked if they can perform their daily activities. This question is part of the EQ-5D-5L questionnaire, which has been shown to have good known-group validity [37,50-52].

Self-care: In addition to daily activities, 1 item of the EQ-5D-5L also assesses self-care [37]. Participants will be asked if they are capable of performing self-care activities.

Dependency: Participants will be asked if they are dependent on other people in their daily lives. This is a newly formulated question.

Return to work or school: This will be assessed by the adjusted International Consortium for Health Outcomes Measurement return to work or school instrument at 6 and 12 months after discharge [38]. Participants will be asked about their daily occupation, with the following response options, (1) “I work 36 hours or more per week,” (2) “I work 35 hours or less per week,” (3) “I am job searching,” (4) “I do not have a paid job (I go to school or am studying),” (5) “I do not have a paid job (retired, volunteer, stay-at-home occupation),” (6) “I cannot work because of my burn injury,” and (7) “I cannot work due to other health issues.” Furthermore, they will be asked if their job has been adjusted since their burn injury (eg, physical adjustments or reduced working hours), with “yes” or “no” as response options. Finally, they will be asked, if relevant, how long after their burn injury they have started working or studying again.

Patient-centeredness: The overall experience of care will be assessed using the Dutch translation of the subscale “patient-centeredness” of the American Consumer Assessment of Health Plan Survey, which consists of 5 questions [39,53]. This scale has been validated for use in the Dutch population and shows good internal consistency (Cronbach α=0.90) [38]. Participants will be asked if they feel listened to by their health care professional, if sufficient time is dedicated to their concerns, if they feel health care professionals take them seriously, if the health care professionals are able to appropriately explain things, and their level of trust in the health care professional’s competence.

##### Health Care Professional Level Outcomes – Secondary Outcome Measures

Self-management support skills: The self-management support skills of the participating health care professionals will be assessed using the Self-Efficacy and Performance in Self-management Support (SEPSS) instrument [40]. The aim of the instrument is to assess self-efficacy and performance in self-management support. The six subscales of the SEPSS are based on the 5 A’s model, (1) assess, (2) advise, (3) agree, (4) assist, (5) arrange, and (6) overall competencies. This questionnaire shows good internal consistency (Cronbach α=0.96) as well as a good test-retest ICC (ICC=0.95).

Need-supportive counseling: The self-management support skills of the participating health care professionals will be objectively evaluated in the preimplementation and postimplementation phases, via video observations using the Coding and Observing Need-Supportive Counseling in Chronic Care Encounters (COUNSEL-CCE) [41]. The survivors of burn injuries and health care professionals who will be filmed will be asked to give consent. Each health care professional will be filmed at least once during the preimplementation and postimplementation phases. The aim of the instrument is to observe need-supportive and need-thwarting counseling in chronic care encounters. The COUNSEL-CCE consists of 44 items divided into nine scales of behavioral approaches, namely (1) participative, (2) attuning, (3) guiding, (4) clarifying, (5) demanding, (6) domineering, (7) abandoning, (8) awaiting, and (9) relatedness supportive reciprocity. The procedure for the observation is the observation of the interaction between the professional and the patient during a 5-minute interval, coding of the observed interval, and observing the same interval for a second time to code any aspects missed. Before scoring the video recordings, the researchers will be trained in the use of the manual, which is provided by the developers of the instrument.

In addition to the COUNSEL-CCE, the video recordings will also be evaluated using the Nurse-Child Interaction Taxonomy (NCIT) [42]. Although this taxonomy was originally developed to improve nurse-child communication, it can also be used to systematically document the interaction between nurses and adult patients. The NCIT consists of 16 elements divided into three main patterns, (1) being considerate, (2) attuning oneself, and (3) procedural intervention.

##### Cost-Effectiveness

The costs will be calculated from a societal perspective. The total costs of burn care will be calculated separately for each survivor of burn injuries in the preimplementation and postimplementation phases. The included cost categories will be health care costs and patients’ costs. Health care costs consist of burn center stay costs, treatment costs, clinical burn consultation costs, and outpatient burn care costs. Patient costs include travel costs, informal care costs, and productivity costs.

To evaluate the cost-effectiveness of the intervention, the complete EQ-5D-5L questionnaire will be used to compare the effectiveness in the control and intervention groups. The EQ-5D-5L questionnaire consists of 5 dimensions (mobility, self-care, daily activities, pain or discomfort, and anxiety or depression) and the EQ visual analog scale [37,50,54].

### RE-AIM: Adoption

Adoption of the intervention will be measured at the staff and setting level. At the staff level, we will assess whether the intervention is adopted by conducting CFIR-informed interviews with burn physicians, nurse practitioners, and aftercare nurses, by asking if they use the BreeZe intervention in their daily work. These interviews will provide working hypotheses to explain the degree of individual intervention adoption. At the setting level, we will assess whether the BreeZe intervention is used within an individual burn center.

### RE-AIM: Implementation

The implementation of BreeZe will be evaluated by assessing the intervention’s fidelity to the core components of BreeZe (eg, goal setting, action planning, and motivational interviewing), implementation fidelity to the implementation strategies (eg, completing the e-learning, attending the training, and being exposed to the additional ERIC implementation strategies), and the adaptations made to BreeZe or to the burn center (eg, workflows).

To gain insight into the intervention fidelity to the core components, health care professionals will be asked to document in patient records when they have used certain components. A convenience sample will be taken by assessing patient records to investigate how often the core components were used.

To gain insight into the implementation fidelity to the implementation strategies, each burn center will be asked to keep track of the completion of the implementation plan, including the staff attendance to the training plan and their e-learning completion rate.

To gain insight into the adaptations made to BreeZe or to the burn center (eg, workflows), adaptations will be documented using the Framework for Reporting Adaptations and Modifications-Enhanced (FRAME) during the postimplementation phase [43].

In addition to assessing the degree of adoption, the CFIR-informed interviews with burn physicians, nurse practitioners, aftercare nurses, and burn center managers will also be used to provide working hypotheses to explain implementation success or failure.

### RE-AIM: Maintenance

Maintenance is assessed at the staff level and setting level [26,27]. At the staff level, maintenance will be assessed by using the health care professionals’ self-report of continued use using the Provider Report of Sustainment Scale (PRESS) at 6 and 12 months post implementation [44]. The PRESS measure is a brief, reliable, and valid 3-item measure that is both pragmatic and usable across different evidence-based practices, provider types, and settings. The PRESS captures health care professionals’ reports of continued use of an intervention in daily practice. In addition, health care professional level maintenance will also be assessed by using the health care professionals’ self-efficacy and performance using the SEPSS-36 at 12 months post implementation [40].

At the setting level, maintenance will be assessed via the Clinical Sustainability Assessment Tool (CSAT) [45]. The CSAT is a brief and reliable instrument consisting of 35 items within 7 domains to assess an institution’s capacity for maintaining a clinical practice. These domains are (1) engaged staff and leadership, (2) engaged stakeholders, (3) organizational readiness, (4) workflow integration, (5) implementation and training, (6) monitoring and evaluation, and (7) outcomes and effectiveness. CSAT development and testing demonstrated excellent internal consistency and several trends toward discriminant validity [45].

In addition to the questionnaires, the CFIR-informed interviews with burn physicians, nurse practitioners, aftercare nurses, and burn center managers will provide working hypotheses to explain the degree of individual (health care professional) and setting-level maintenance.

### Participant Timeline

The participant timelines illustrated in Table 2 (for survivors of burn injuries) and Table 3 (for health care professionals) outline the different assessment time points of the study.

**Table 2.** Timeline for participating survivors of burn injuries: enrollment, interventions, and assessments.

	Study period – survivors of burn injuries				
	Enrollment	Allocation	Postallocation		Closeout
	Before discharge	At discharge	T1 (2 weeks postdischarge)	T2 (6 months postdischarge)	T3 (12 months postdischarge)
Enrollment					
Eligibility screen	✓				
Informed consent	✓				
Allocation		✓			
Interventions					
Control (usual care)^a^			✓	✓	✓
BreeZe aftercare^a^			✓	✓	✓
Assessments					
Demographics		✓			
Self-management skills (PiH^b^) [34]			✓	✓	✓
Self-regulation (SeRA^c^) [35]			✓	✓	✓
Participation (MeEAP^d^) [36]			✓	✓	
Daily activities (EQ-5D-5L) [37]			✓	✓	✓
Self-care (EQ-5D-5L) [37]			✓	✓	✓
Dependency			✓	✓	✓
Return to work or school (ICHOM^e^) [38]				✓	✓
Patient centeredness (CAHPS^f^) [39,53]				✓	
Quality of life (EQ-5D-5L) [37]			✓	✓	✓

a Duration of usual or BreeZe aftercare depends on the personal trajectory of the survivor of burn injuries.

b PiH: Partners in Health scale.

c SeRA: Self-Regulation Assessment.

d MeEAP: Measure of Experiential Aspects of Participation.

e ICHOM: Adjusted International Consortium for Health Outcomes Measurement.

f CAHPS: Consumer Assessment of Health Plan Survey.

**Table 3.** Timeline for participating health care professionals: enrollment, interventions, and assessments.

	Study period – health care professionals				
	Enrollment	Allocation	Postallocation		Closeout
	Preimplementation phase	Implementation phase	T1 (3 months postimplementation phase)	T2 (6 months post implementation phase)	T3 (12 months postimplementation phase)
Enrollment					
Eligibility screen	✓				
Informed consent	✓				
Allocation		✓			
Interventions					
Training		✓			
Assessments					
Demographics	✓				
Self-management support skills (SEPSS-36^a^) [40]	✓		✓	✓	✓
Need-supportive counseling (COUNSEL-CCE^b^ and NCIT^c^) [41,42]	✓		✓		✓
Maintenance (PRESS^d^) [44]				✓	✓
Maintenance (CSAT^e^ ) [45]			✓		✓
CFIR^f^-informed interviews				✓	

a SEPSS-36: Self-Efficacy and Performance in Self-Management Support Questionnaire.

b COUNSEL-CCE: Coding and Observing Need-Supportive Counselling in Chronic Care Encounters.

c NCIT: Nurse-Child Interaction Taxonomy.

d PRESS: Provider Report of Sustainment Scale.

e CSAT: Clinical Sustainability Assessment Tool.

f CFIR: Consolidated Framework for Implementation Research.

### Sample Size

To estimate sample size, we used a power calculation based on our primary outcome, which is self-management skills measured by the PiH questionnaire. As no minimum clinically important difference is established in the literature, 2 burn care nurse practitioners examined the PiH and considered an improvement of 8 points as clinically relevant. Based on this face-validity score and a previous dataset with PiH data from survivors of burn injuries collected with the Burn Centers Outcomes Registry the Netherlands (BORN), a mean difference of 8 points between the control and intervention group is feasible. As there is no validated stepped-wedge–specific sample size calculator for this outcome, we used a pragmatic approximation based on this predefined clinically relevant difference we established. In addition, as there is no data available on potential ICCs and we have a small number of burn centers, we will take center difference later on into account by adding 2 dummy variables (with center 1 being the reference center) in our analyses [55]. For the sample size calculation, we used the online sample size calculator of the University of British Columbia [56]. We aim to obtain 80% power with a 2-sided significance level of 0.05. Due to the stepped-wedge design of our study, our allocation rate for the control group:intervention group will be 0.625:1. With an adjustment of 20% for dropouts, missing data, and fixed time effects taken into account, we calculated that 57 participants in the control group and 89 participants in the intervention group will be sufficient. This amount (n=146) is deemed sufficient for (multilevel) regression analyses using the MIwiN statistical program, version 3.10 [57].

### Recruitment

As of July 15, 2022, all survivors of burn injuries receiving burn care in one of the 3 centers are asked by a nurse practitioner or aftercare nurse to complete the routinely collected value-based health care burns core set for adult survivors of burn injuries as part of the Dutch Burn Repository R3 and the BORN [58]. In addition to the routinely collected core set data, survivors of burn injuries who participate during the pre- and postimplementation phases of this study will be asked to complete additional questionnaires. A nurse practitioner or aftercare nurse will introduce the study to eligible patients and provide a patient information letter. After an appropriate consideration time (2 d), a member of the center’s research team will answer any questions about the study and take informed consent. By involving the nurse practitioners and aftercare nurses, all eligible survivors of burn injuries will be reached.

### Assignment of Interventions

#### Sequence Generation

We believe that a burn center’s enthusiasm to participate in the intervention serves as a preliminary indicator of its organizational readiness. Organizational readiness is a crucial determinant that influences the implementation success or failure [59]. Hence, we decided to determine the order of commencement based on our interpretation of the burn centers’ organizational readiness.

Patients will be recruited in consecutive order in the preimplementation phase as well as in the postimplementation phase. In principle, all health care professionals involved in burns aftercare will be recruited.

#### Blinding, Concealment Mechanism, and Implementation

Blinding is not feasible due to the nature of the intervention. As blinding is not feasible, no procedures for unblinding are necessary. Furthermore, due to the lack of blinding and randomization, no concealment mechanism is necessary. The sequence allocation of the centers has been chosen in deliberation with the BreeZe project team. In each center, 2 or 3 specialized researchers are responsible for enrollment and data collection.

### Data Collection and Management

#### Data Collection Methods

##### Survivors of Burn Injuries

Assessment time points are at discharge, 2 weeks after discharge, and 6 and 12 months after discharge, as pictured in Table 2. Questionnaires at 2 weeks after discharge and 12 months are part of the routinely collected value-based health care burns core set, from the BORN [58]. Participants receive the questionnaires by email. In cases where patients do not have an email address or access to a computer, the questionnaire can be completed by telephone interview or on paper. Using the Dutch Burn Repository R3, the characteristics sex, age, and total burned body surface area will be collected from all eligible survivors of burn injuries during the study period.

##### Health Care Professionals

Assessment time points with health care professionals are preimplementation, and at 3, 6, and 12 months post implementation, as pictured in Table 3. They will receive the questionnaires via email or on paper, distributed by the responsible researcher.

Consultations that are eligible for video observations will be discussed and selected by the research team and health care professionals. Consultations will be eligible if they qualify as an aftercare consultation. Survivors of burn injuries will be informed and asked by a member of the research team 1 to 5 days before their appointment if they agree that their encounter is filmed. Before the start of the consultation, the researcher sets up the video camera and starts the recording. Then, the researcher leaves the room, and the health care professional and the survivor of burn injuries will start the consultation. Both the health care professional and survivor of burn injuries will be visible in the recording. At the end of the consultation and when the health care professional and survivor of burn injuries have left the room, the researcher will stop the recording and transfer the recording directly from the camera to the specialized research file. After the transfer, the recording will be directly deleted from the video camera.

The interviews will be carried out by the main researcher. Beforehand, a CFIR-informed topic guide will be developed. The interviews will be recorded with a tape recorder and will last approximately 45 minutes.

### Plans to Promote Participant Retention and Complete Follow-Up

#### Survivors of Burn Injuries

To promote completion of the follow-up questionnaires, a researcher will remind participants a maximum of 2 times if they have not completed the questionnaires. These reminders can be an email or a phone call. We will adhere to an intention-to-treat approach. If treatment at the burn center is concluded before the survivor of burn injuries has been through all the BreeZe phases, the questionnaires will continue to be sent, except when a survivor of burn injuries withdraws their consent to participate.

#### Health Care Professionals

Health care professionals will be reminded by the responsible researchers to complete questionnaires, to select consultations for the video observations, and to set a date for the interviews.

### Data Management

The responsible researcher of each center will save data in password-protected Microsoft Excel files, which are stored on a protected working drive. Passwords of the files are only known by the involved researchers. Data will be stored for 15 years according to the Dutch Personal Data Protection Law.

#### Survivors of Burn Injuries

Eligible survivors of burn injuries will be identified by the nurse practitioners or aftercare nurses, which will be communicated with the research team. For entry in the password-protected eligibility file, the characteristics documented are name, patient number, date of admission, date of surgery, date of discharge, and participation (yes or no, with reason if no).

Informed consent will be taken on paper and stored in a locked cupboard at the research office of each burn center. After the informed consent has been taken, each survivor of burn injuries will be assigned a unique study code which will be entered in a password-protected key data file in Excel containing key, name, and patient number.

Questionnaires will be sent out via Lighthouse QuestManager. Data will be extracted in an Excel file and then transported into IBM SPSS (version 25 or higher versions).

#### Health Care Professionals

Information on eligible health care professionals will be included in a password-protected eligibility file in Excel, which will be stored on a protected working drive. The password of the eligibility file is only known by the involved researchers. For entry in the eligibility file, the characteristics of eligible health care professionals that are documented are name, years of experience within the profession (divided into categories), and participation (yes or no).

Informed consent will be taken on paper and stored in a locked cupboard at the research office of each burn center. After taking informed consent, each health care professional will be assigned a unique study code in a password-protected key data file in Excel.

Questionnaires will be sent out on paper or via Castor [60]. Data from paper questionnaires will be imported into an Excel file and then transported into SPSS or MLwiN (Center for Multilevel Modelling, University of Bristol) [57]. Video recording files will be directly transferred from the video camera to the research working drive. Interview recordings will be directly transferred from the tape recorder to the research working drive, and then transported into Atlas.TI.

### Statistical Methods

#### Statistical Methods for Primary and Secondary Outcomes

##### Overview

All quantitative analyses will be conducted using MLwiN 3.10 for nested data or IBM SPSS Statistics [57]. A *P* value <.05 will be considered statistically significant. For all outcomes, descriptive statistics will be used to present demographics and outcomes, using frequencies (%) for categorical variables, and median and IQR (if nonnormally distributed) or mean and SD (if normally distributed) for continuous variables.

##### RE-AIM: Reach

To examine if participating survivors of burn injuries are representative of the general survivor of burn injuries population, baseline demographics of participating survivors of burn injuries and nonparticipating survivors of burn injuries will be compared using chi-square tests for categorical data, and Mann-Whitney or independent *t* tests for numerical data.

##### RE-AIM: Effectiveness

###### Effectiveness on Health Outcomes of Survivors of Burn Injuries – Coprimary Outcome Measure

To examine if participants in the control and intervention groups are comparable on baseline demographics and clinical outcomes, chi-square tests will be used for categorical data, and Mann-Whitney or independent *t* tests will be used for numerical data. To test the effectiveness of BreeZe on health outcomes of survivors of burn injuries, we will conduct (multilevel) regression analyses for repeated measurements, with measurement occasions nested in patients. Variables that might explain differences between measurement occasions (eg, time since injury, time since discharge, season, and time since intervention started) or patient (eg, age and sex) will be tested. We expect that the variance between measurement occasions will be largely explained at the level of patients. To account for the difference between centers, we will add 2 dummy variables (with center 1 being the reference center) to these analyses [55].

In addition, as control and intervention participants are enrolled in different calendar periods in different centers due to the stepped-wedge design, we will test in the (multilevel) regression analyses whether time can best be modeled continuously (since discharge) or as a categorical variable. The latter is interpreted more easily and is more often used in clinical settings, but does increase the degrees of freedom in the model. We will also document major changes in burn care organization, staffing, or parallel initiatives during the study and consider these in sensitivity analyses and in the interpretation of our findings.

The primary data analyses will be conducted on the data of the PiH questionnaire. In the multilevel analyses, the patient’s PiH score at a certain moment is the dependent variable. In addition, we will use three different approaches to provide a comprehensive evaluation of the intervention’s impact. First, for each group, the number and percentage of participants who have improved by at least 8 points on their self-management skills will be calculated, and the difference between groups will be tested with a chi-square test. We consider an improvement of 8 points a clinically relevant score, based on a face validity assessment by burn care nurse practitioners.

Second, to examine whether greater improvement is achieved by individuals with low compared with high baseline scores, we will create 2 groups based on the median score. Besides a baseline effect and an intervention effect, an interaction effect will be tested.

Third, for each participant, we will tally the number of PiH questions where they score 6 or higher, as such a score will be considered clinically satisfactory. This sum score will range from 0 to 12 and will also be used as a dependent variable in a regression analysis with group and time as possible predictors. Our hypothesis is that there will be an increase in this sum score, which will be greater in the intervention group compared to the control group.

###### Effectiveness on Health Outcomes of Survivors of Burn Injuries – Secondary Outcome Measures

For the other outcome measures, we will perform similar multilevel analyses, testing the effects of baseline scores and intervention. In addition, we will calculate correlation coefficients to explore the association between the PiH and the SeRa. Correlation coefficients will be interpreted as negligible correlation (0‐0.3), low correlation (0.3‐0.5), moderate correlation (0.5‐0.7), high correlation (0.7‐0.9), or very high correlation (0.9‐1.0) [61]. This method will allow us to explore the potential correlation between the constructs of self-management skills and self-regulation skills.

###### Health Care Professionals Level Outcomes – Secondary Outcome Measures

To evaluate the effect of the training on self-management support skills, we will use the data from the SEPSS-36 in a 2-level regression model with measurement occasions within a health care professional.

For the video observations, the video recordings will be broken down into 5-minute intervals. Each interval will be coded independently by 2 researchers according to the NCIT and COUNCEL-CCE coding manuals. Results will be presented with descriptive statistics. Differences in observations through time will be tested with (multilevel) regression analyses for repeated measures.

###### Setting Level Outcomes – Cost-Effectiveness

Burn care costs will be calculated by multiplying the volumes of health care used by the corresponding unit prices. The unit prices were derived from the Dutch guidelines [62] and previous studies [63-65]. The unit prices of previous studies will be updated by the inflation correction of the current financial year. Substantive additions, for instance, patients’ costs will be based on previous studies and current care.

Utility values will be obtained from each patient with and without the BreeZe intervention by using the EQ-5D-5L questionnaire. The utility scores per domain range between 0 and 1, whereas 0 is death and 1 is perfect health. The total utility score per patient will be calculated. Quality-adjusted life years will be calculated by multiplying the length of time spent in a particular health state by its associated utility score.

### RE-AIM: Adoption

At the staff level, adoption will be assessed with the results of the PRESS at 6 months post implementation, presented using descriptive statistics. At the setting level, adoption will be assessed by conducting CFIR-informed interviews with all participating health care professionals. These interviews will be transcribed verbatim and analyzed using a thematic content analysis using ATLAS.ti. Furthermore, 2 researchers will independently code each interview. Then, the researchers organize codes into categories and identify key themes that reflect adoption-related data.

### RE-AIM: Implementation

To assess intervention fidelity, health care professionals are asked to document in patient records if, when, and which components of the BreeZe intervention they have used (eg, the Self-management web, goal setting, and action planning). This documentation will be used to assess how often certain components are used.

To assess implementation fidelity to the implementation strategies, the results of the implementation plan will be analyzed and presented with descriptive statistics (eg, completing the e-learning, attending the training, and being exposed to the additional ERIC implementation strategies).

Furthermore, to gain insight into the adaptations made to BreeZe or to the burn center (eg, workflows), we analyze the adaptations that have been documented during the study. These results will be schematically presented and summarized in text.

### RE-AIM: Maintenance

At the individual health care professional level, maintenance will be assessed by using the results of the SEPSS-36 at 12 months post implementation (refer to Health Care Professional Level Outcomes – Secondary Outcome Measures). Furthermore, the PRESS outcomes at 12 months post implementation will be presented using descriptive statistics.

At the setting level, maintenance will be assessed by using the results of the CSAT questionnaire. Results of each center will be separately presented. First, descriptive statistics will be presented for both the total score as well as the scores of each subdomain. Second, an independent *t* test or a Mann-Whitney test will be used to assess the difference in results over time.

### Interim Analyses

No interim analyses have been defined, due to the low anticipated incidence of adverse events in both the control and intervention group.

### Methods for Additional Analyses

Our analyses, as described above, are based on an intention-to-treat approach. If survivors of burn injuries did start with the BreeZe intervention but did not complete it as intended, this will be documented. In case this occurs, we will perform additional analyses conforming to this protocol using only the subgroup of survivors of burn injuries who received and/or used at least 2 BreeZe components (eg, workbook and self-management web).

### Methods in Analysis to Handle Protocol Nonadherence and Any Statistical Methods to Handle Missing Data

All analyses will be conducted based on the intention-to-treat approach. This means that all included survivors of burn injuries will be included in the analysis according to their assigned groups, regardless of their treatment adherence. This approach requires complete data for all participating survivors of burn injuries, and we will strive to minimize missing data throughout the study by keeping track of the completion of the questionnaires.

Missing data will be replaced with the value 999. To establish whether missing data is missing completely at random, missing at random, or missing not at random, we will use descriptive tables, frequencies, and correlation plots to identify potential patterns. If data are missing randomly, a maximum likelihood estimation model will be used to estimate parameters based on all available data. An advantage of performing multilevel analyses for nested data in patients is that these can be performed even if a whole measurement occasion is missing. However, then the missingness should be assumed to be random. If data are not missing randomly, we will conduct sensitivity analyses to examine how different assumptions about the missing data affect the results and evaluate the potential impact of the missing data on our conclusions. Finally, we will document the extent of missing data and the reasons for missingness.

### Oversight and Monitoring

#### Composition of the Coordinating Center and Trial Steering Committee

This study will be coordinated by the research department of the burn center at the Martini Hospital. The primary research group will consist of the study principal investigator (MKN), a burn physician (SMHJSJ), a postdoctoral researcher (SJGG), and a PhD student (SLB). The latter will be responsible for running the overall study-related activities and the day-to-day organizational support at the Martini Hospital. At the other 2 burn centers, a local researcher will be the study coordinator and responsible for day-to-day organizational support and data management. These local study coordinators work in close collaboration with the coordinating group from the Martini Hospital, meeting at least once a month.

#### Data Monitoring Committee

This study does not establish a data and safety monitoring board, as the BreeZe intervention is regarded as low-risk and does not present any significant safety concerns.

#### Adverse Event Reporting and Harms

As the BreeZe intervention is regarded as low-risk, we do not anticipate any adverse events. Any unanticipated effects of the intervention will be documented and reported to the local Medical Ethics Committees of the Martini Hospital, Red Cross Hospital, and the Maasstad Hospital.

#### Trial Monitoring

This study will be supervised by the local Medical Ethics Committees of the Martini Hospital, Red Cross Hospital, and the Maasstad Hospital.

#### Patient and Public Involvement

The development of the BreeZe intervention was guided by a strong commitment to patient and public involvement. The research team included 2 patient experts as integral members of the team. One patient expert was engaged from the beginning of the project in 2022, and the second patient expert joined the research team in February 2023. They participated in monthly development sessions, which were facilitated by the lead researcher (SJGG) using a structured consensus-building approach as described by Leising and colleagues [66], emphasizing partnership-based collaboration. Within this process, the patient experts were positioned not merely as consultees but as cocreators, encouraged to actively contribute their experiential knowledge and perspectives.

Following the completion of the initial draft of the intervention, the research team, including the patient experts, continued to meet on a monthly basis. The focus of these sessions shifted toward implementation planning and the design of the evaluation study. The initial methodological framework was drafted by a postdoctoral researcher (SJGG) and a PhD candidate (SLB); however, systematic input and iterative feedback were sought from the patient experts to ensure that the design was responsive to patient needs and priorities. The selection of outcomes was primarily informed by the value-based health care burns core set, which itself was developed with substantive patient involvement to ensure the inclusion of outcomes and quality indicators most relevant to survivors of burn injuries [58].

Beyond development and evaluation, the role of the patient experts extends into dissemination. In collaboration with the Dutch Burn Survivors Association, they will contribute to knowledge translation activities, including advising on the accessibility and relevance of dissemination outputs and facilitating reach through their established networks.

### Ethical Considerations

This study follows the procedures in accordance with the World Medical Association’s Declaration of Helsinki and has been approved by the local Medical Ethical Committees of the Martini Hospital (reference 2024‐033 and 2024‐48), the Red Cross Hospital (references 24.010 and 24.018), and Maasstad Hospital (reference L2024046 and L2024086). All participating survivors of burn injuries and health care professionals will provide written informed consent. Participants are reassured that participation is voluntary and that withdrawal is possible at any time. No identifying information will be published. No compensation is available for participants.

### Protocol Amendments

In the event of any protocol amendments, the local Medical Ethics Committee will be notified directly and asked for approval. After obtaining approval, the amendments will be communicated by the research team to all involved parties.

### Consent

#### Survivors of Burn Injuries

As of July 15, 2022, survivors of burn injuries receiving burn care in 1 of 3 centers are asked by their health care professional to participate and complete the routinely collected value-based health care burns core set for adult survivors of burn injuries, as part of the Dutch Burn Repository R3 and the BORN [58]. In addition to these routinely collected data, a member of the research team will ask eligible survivors of burn injuries to complete additional questionnaires. This researcher will take informed consent if a survivor of burn injuries is willing to participate. Informed consent will be taken on paper and stored in a locked cupboard at the research office of each burn center. After the informed consent has been taken, each survivor of burn injuries will be assigned a unique study code, which will be entered in a password-protected key data file in Excel containing key, name, and patient number. Participants are reassured that participation is voluntary and that withdrawal is possible at any time.

#### Health Care Professionals

Informed consent from eligible health care professionals will be taken by a member of the research team of each burn center. Informed consent will be taken on paper and stored in a locked cupboard at the research office of each burn center. After taking informed consent, each health care professional will be assigned a unique study code in a password-protected key data file in Excel. Participants are reassured that participation is voluntary and that withdrawal is possible at any time.

#### Additional Consent

Enrolled health care professionals will participate in video observations of aftercare consultations to collect outcome data. Survivors of burn injuries will be informed and asked beforehand by a member of the research team if they agree to be filmed. If they agree, these survivors of burn injuries will sign an additional informed consent. Participants are reassured that participation is voluntary and that withdrawal is possible at any time.

All consent forms used in this study are available on request to the corresponding author. Every author has approved the final manuscript in its submitted form and is willing to take responsibility for all aspects of the research.

### Confidentiality

#### Survivors of Burn Injuries

Eligible survivors of burn injuries will be identified by their practicing health care professional, which will be communicated with the main researchers. Then, researchers can enter patient records to identify demographic characteristics for the eligibility file. After enrolling in the study, participants are assigned an individual study code (key). For further data collection, only the study code will be assigned to the data, thereby maintaining confidentiality. Only the main researchers have access to the key document with participants’ names. No identifying information will be published.

#### Health Care Professionals

Due to the small number of health care professionals in our study, we will adjust the data collection of the demographics. To maintain confidentiality, age and years of experience will be collected in categories, with 5-year steps. In addition, all health care professionals will be assigned a unique study code (key). For further data collection, only the study code will be assigned to the data. Only the main researchers have access to the key document with participants’ names. No identifying information will be published.

### Ancillary and Posttrial Care

The aim of the implementation of BreeZe during the implementation phase is to integrate BreeZe into daily practice. Necessary materials, such as laminated self-management webs and workbooks, are provided by the researchers to each burn center, which are allowed to keep after the implementation phase. No other forms of compensation are available for participants. Plans to ensure continuity of the BreeZe intervention are currently in progress.

### Dissemination and Data Availability

The results of the study will be disseminated in scientific articles for publication in peer-reviewed journals. Both positive and negative results will be reported. Furthermore, we aim to present the results at national and international scientific conferences and other relevant meetings with stakeholders, including patient associations. Finally, the results will be disseminated via social and published media.

## Results

This research is part of a larger project, which was funded in January 2022. Participant recruitment ended on June 30, 2025. In total, 42 participants are included in the control group, and 53 participants are included in the intervention group. Follow-up data collection is currently ongoing and will end in July 2026. Data analysis will start in August 2026, and the results are expected to be published in May 2027.

## Discussion

### Principal Findings

The aim of this study protocol was to describe the study that evaluates the effectiveness and implementation of the BreeZe intervention, a self-management support intervention that aims at improving self-management skills of survivors of burn injuries.

### Methodological Considerations

One of the key strengths of this study protocol is the use of the well-established implementation science determinant framework, CFIR, in our implementation approach [25]. In addition to basing our intervention strategies on evidence-based techniques (eg, motivational interviewing), the implementation approach plays a crucial role in determining intervention success or failure [67]. The CFIR is a determinant framework designed to facilitate understanding and explanation of determinants (ie, facilitators and barriers) influencing implementation outcomes [68]. By prospectively identifying these determinants, we can use the appropriate implementation strategies to optimize the implementation process and ensure that all relevant factors, such as context, stakeholders, and resources, are taken into account [69,70].

Another key strength is the use of the implementation science evaluation framework, RE-AIM, in our outcome assessment. RE-AIM is an evaluation framework that helps to specify and assess aspects of implementation to determine overall success in terms of both intervention effectiveness and implementation [26,27]. Without specifying and assessing aspects of implementation, it is difficult to determine whether an intervention’s success or failure is due to the intervention itself or how it was implemented [71].

The use of the RE-AIM framework also signifies that we are conducting a hybrid effectiveness-implementation study. Hybrid studies assess both the effectiveness of an intervention on health outcomes of a population and assess the effect of an implementation approach on implementation outcomes [23,24]. These studies are well-advocated as they can distinguish whether findings originate from the implementation process or the intervention itself, and they have the potential to accelerate the translation of research into clinical practice [23,72-74]. However, the combination of both effectiveness and implementation measures makes these hybrid studies highly extensive, which can pose challenges in execution due to resource constraints, logistical considerations, and time limitations [74,75]. Given the resources and time available, we have designed the most rigorous and robust study possible, ensuring that we included sufficient outcomes and outcome measures.

A last methodological consideration in designing this study is our aim to strike a careful balance between internal and external validity, ensuring that the study is both generalizable to different burn care settings and sufficiently rigorous and robust to provide reliable answers to our research questions. While a parallel cluster randomized design is often considered the gold standard for minimizing bias and ensuring strong internal validity, we opted for a nonrandomized stepped-wedge design [24,76,77]. A parallel cluster randomized design, in which all clusters are randomly assigned to either the intervention or control condition, is often challenging in real-world settings, as it requires immediate and simultaneous implementation across all centers. Implementation success strongly influences the outcomes of an intervention, and whether you achieve implementation success is strongly dependent on organizational readiness [59]. Also, a parallel cluster randomized design is potentially less acceptable for stakeholders, as at least 1 cluster acts as the control group, meaning they are not exposed to the potential benefits of the intervention [24,78]. In contrast, a nonrandomized stepped-wedge design allows for a staggered implementation, where all centers eventually receive the intervention but at different time points and is logistically feasible [24]. Hence, to increase stakeholder acceptability and organizational readiness, we made the choice not to randomize. However, a nonrandomized stepped-wedge design carries a greater risk of bias, particularly due to lack of randomization, time-related confounders, and potential differences between early and late adopters [79,80]. To control for these, we took fixed-time effects into account in our sample size calculation [81].

Furthermore, while ensuring high internal validity is essential for accurately assessing the effectiveness of the intervention and effects of the implementation approach, it may limit the generalizability of the findings to real-world settings [82-84]. To enhance external validity, we decided to apply the same inclusion criteria for this study as those used for the patient-reported outcomes measures of the BORN, ensuring that the BreeZe intervention potentially reaches a large proportion of survivors of burn injuries in the Netherlands, and that the results are more generalizable to survivors of burn injuries populations in other countries. Finally, by integrating the BreeZe intervention into routine clinical practice, our study reflects real-world conditions, further enhancing its external validity.

## Supplementary material

10.2196/86671 Multimedia Appendix 1 Definitions of the Reach, Effectiveness, Adoption, Implementation, and Maintenance dimensions, its related research questions, outcome measures, and data sources.

10.2196/86671 Checklist 1 SPIRIT checklist.

## Abbreviations

BORN: Burn centers Outcome Registry Netherlands
BreeZe: Brandwonden en Zelfmanagement ("Burns and Self-management")
CFIR: Consolidated Framework for Implementation Research
COUNSEL-CCE: Coding and Observing Need-Supportive Counseling in Chronic Care Encounters
CSAT: Clinical Sustainability Assessment Tool
ERIC: expert recommendations for implementing change
FRAME: Framework for Reporting Adaptations and Modifications-Enhanced
ICC: intraclass correlation coefficient
NCIT: Nurse-Child Interaction Taxonomy
PiH: Partners in Health
PRESS: Provider Report of Sustainment Scale
RE-AIM: Reach, Effectiveness, Adoption, Implementation, and Maintenance
SEPSS: Self-Efficacy and Performance in Self-management Support
SeRA: Self-Regulation Assessment
SMART: Specific, Measurable, Attainable, Relevant, and Time-based
ZENN: ZElfmanagement Na Niertransplantatie (‘Self-management after kidney transplantation’)
