# Supplementary material for: Self-Management Support After Burns: Protocol for a Multicenter, Stepped-Wedge Hybrid Type II Effectiveness-Implementation Study
Source: JMIR Res Protoc. 2026 Mar 16;15:e86671. doi: 10.2196/86671 (PMC12991193; doi:10.2196/86671)
Supplement: Multimedia Appendix 1 [file resprot-v15-e86671-s001.docx]

*Table S1. The RE-AIM dimension definitions and different research questions posed in this study*

| **Outcome** | **Definition** | **Research questions** |
| --- | --- | --- |
| Reach | The absolute number, proportion, and representativeness of individuals/centres who are willing to participate in a given initiative | 1. How many participants received the BreeZe intervention during the study period? 2. Is there a difference in demographic characteristics of participants in the intervention group and control group? |
| Effectiveness | The impact of an intervention on important outcomes, including potential negative effects, quality of life, and economic outcomes | Primary research question:   1. What are the differences in participants’ self-management skills pre- and post-intervention? 2. What are the differences in participants’ self-management skills between participants in the control and experimental period? |
|  |  | Secondary research questions:   1. What are the differences in participants’ self-regulation skills pre- and post-intervention? 2. What are the differences in participants’ self-regulation skills between participants in the control and experimental period? 3. What is the difference between participants’ perceived independence in the control and experimental period? 4. What is the difference between participants’ self-care abilities in the control and experimental period? 5. What is the difference between participants’ abilities to carry out daily activities in the control and experimental period? 6. Does the BreeZe intervention increase the amount of burn survivors who return to their work/study within a year? 7. Is the BreeZe intervention more patient-centred than usual care? 8. Does the BreeZe intervention improve healthcare professionals’ self-management support skills? 9. Does the BreeZe intervention improve healthcare professionals’ self-efficacy in providing self-management support? |
| Adoption | The absolute number, proportion, and representativeness of settings and intervention agents who are willing to initiate a program | 1. How many Burn Centres participated in the BreeZe intervention? 2. What percentage of healthcare professionals who were invited to participate, did participate in the BreeZe intervention? 3. What are healthcare professionals’ experiences and appreciation of the self-management intervention? |
| Implementation | The intervention agents’ fidelity to the intervention’s protocol, including consistency of delivery as intended, time and cost of the intervention, and program adaptations | 1. How many healthcare professionals completed the training program of the BreeZe intervention? 2. To what extent did the healthcare professionals carry out the BreeZe intervention as described in the protocol? 3. What are the experiences of healthcare professionals regarding the applicability, usability, and acceptability of the BreeZe intervention? 4. What are healthcare professionals’ experiences with the BreeZe intervention? 5. What are the context-bound adaptations that are necessary for each Burn Centre? |
| Maintenance | The extent to which a program or policy becomes institutionalized or part of the routine organizational practices and policies | 1. How many burn survivors were eligible for the BreeZe intervention but did not participate in the first 12 months post-transition phase? 2. What are the Burn Centres sustainability capacities for the BreeZe intervention? 3. What are the long-term attrition rates? 4. To what extent can the BreeZe intervention be maintained within the organisation? |

*Table S2. The RE-AIM dimension definitions are presented together with the measures, and data sources needed to provide answer to the research questions.*

| **Outcome** | **Definition** | **Measure(s)** | **Data source(s)** |
| --- | --- | --- | --- |
| Demographic variables | Characteristics of sample population | **Burn survivor** |  |
|  |  | Sex, age, TBSA^a^, length of stay | Dutch Burn Repository R3 |
|  |  | **Healthcare professional** |  |
|  |  | Age, profession, work experience | Project coordinator records |
| Reach | The absolute number, proportion, and representativeness of individuals/centres who are willing to participate in a given initiative | Eligibility criteria | Screening form |
|  |  | Number of burn survivors who were eligible for BreeZe | Dutch Burn Repository R3 |
|  |  | Number of burn survivors who received BreeZe | Project coordinator records |
|  |  | Reasons for not participating in BreeZe | Screening form |
|  |  | Barriers and facilitators to recruitment | Interviews with healthcare professionals |
| Effectiveness | The impact of an intervention on important outcomes, including potential negative effects, quality of life, and economic outcomes | **Burn survivor** |  |
|  |  | Self-management skills (2 weeks, 6 months to 12 months post discharge) | Partners in Health (PIH) Questionnaire |
|  |  | Self-regulation in the context of burns | Self-Regulation Assessment (SeRA) |
|  |  | Self-care | EuroQol 5D 5L (EQ5D-5L) |
|  |  | Daily activities | EuroQol 5D 5L (EQ5D-5L) |
|  |  | Independency | Newly developed question |
|  |  | Return to work/school | Adjusted International Consortium for Health Outcomes Measurement (ICHOM) return to work/school instrument |
|  |  | Patient centeredness | Subscale ‘patient-centeredness’ of the American Consumer Assessment of Health Plan Survey (CAHPS) |
|  |  | **Healthcare professional** |  |
|  |  | Self-management support skills – self-efficacy | Self-Efficacy and Performance in Self-management Support (SEPSS) instrument |
|  |  | Self-management support skills - performance | Self-Efficacy and Performance in Self-management Support (SEPSS) instrument |
|  |  | Need-supportive counselling | Video analysis using the Coding and Observing Need-Supportive Counselling in Chronic Care Encounters (COUNSEL-CCE) and Nurse-Child Interaction Taxonomy (NCIT) |
|  |  | **Setting level** | |
|  |  | Cost-effectiveness | EuroQol-5D |
| Adoption | The absolute number, proportion, and representativeness of settings and intervention agents who are willing to initiate a program | **Staff level** |  |
|  |  | Staff exclusions (reasons) | Project coordinator records |
|  |  | Percent of staff invited | Project coordinator records |
|  |  | Reasons for (non-)participation | Interviews among healthcare professionals |
|  |  | **Setting level** |  |
|  |  | Setting exclusions (reasons) | Project coordinator records |
| Implementation | The intervention agents’ fidelity to the intervention’s protocol, including consistency of delivery as intended, time and cost of the intervention, and program adaptations | Fidelity | Project coordinator records |
|  |  | Adaptations made to BreeZe as intervention | Project coordinator records |
|  |  | Adaptations made to the BreeZe implementation plan | Project coordinator records |
|  |  | Attendance to training plan | Project coordinator records |
|  |  | E-learning completion rate | Project coordinator records |
|  |  | Factors of influence on implementation | Interviews among healthcare professionals |
| Maintenance | The extent to which a program or policy becomes institutionalized or part of the routine organizational practices and policies | Number of burn survivors who were eligible for BreeZe but did not receive it during the first 12 months post-transition phase | Dutch Burn Repository R3;  Project coordinator records |
|  |  | Long term attrition rates (i.e., not completing session 1 to 4) | Project coordinator records |
|  |  | **Individual – Healthcare professionals** |  |
|  |  | Self-efficacy and performance in providing self-management support (post-implementation to 12 months) | Self-Efficacy and Performance in Self-management Support (SEPSS-36) |
|  |  | Continued use of BreeZe | Provider REport of Sustainment Scale (PRESS) |
|  |  | Factors of influence on sustainability of BreeZe | Interviews with healthcare professionals |
|  |  | **Setting** |  |
|  |  | Burn centres’ sustainability capacity for BreeZe | Clinical Sustainability Assessment Tool (CSAT) |
